# Total knee arthroplasty after high tibial osteotomy. A systematic review

**DOI:** 10.1186/1471-2474-10-88

**Published:** 2009-07-20

**Authors:** Tom M van Raaij, Max Reijman, Andrea D Furlan, Jan AN Verhaar

**Affiliations:** 1 Department of Orthopaedics, Erasmus University Medical Center, Rotterdam, the Netherlands; 2 Department of Orthopaedics, Martini Hospital, Groningen, the Netherlands; 3 Institute for Work & Health, Toronto, Canada; 4 Toronto Rehabilitation Institute, Toronto, Canada

## Abstract

**Background:**

Previous osteotomy may compromise subsequent knee replacement, but no guidelines considering knee arthroplasty after prior osteotomy have been developed. We describe a systematic review of non-randomized studies to analyze the effect of high tibial osteotomy on total knee arthroplasty.

**Methods:**

A computerized search for relevant studies published up to September 2007 was performed in Medline and Embase using a search strategy that is highly sensitive to find nonrandomized studies. Included were observational studies in which patients had total knee arthroplasty performed after prior high tibial osteotomy. Studies that fulfilled these criteria, were assessed for methodologic quality by two independent reviewers using the critical appraisal of observational studies developed by Deeks and the MINORS instrument. The study characteristics and data on the intervention, follow-up, and outcome measures, were extracted using a pre-tested standardized form. Primary outcomes were: knee range of motion, knee clinical score, and revision surgery. The grade of evidence was determined using the guidelines of the GRADE working group.

**Results:**

Of the 458 articles identified using our search strategy, 17 met the inclusion criteria. Fifteen studies were cohort study with a concurrent control group, one was a historical cohort study and one a case-control study. Nine studies scored 50% or more on both methodological quality assessments. Pooling of the results was not possible due to the heterogeneity of the studies, and our analysis could not raise the overall low quality of evidence. No significant differences between primary total knee arthroplasty and total knee arthroplasty after osteotomy were found for knee range of motion in four out of six studies, knee clinical scores in eight out of nine studies, and revision surgery in eight out of eight studies after a median follow-up of 5 years.

**Conclusion:**

Our analysis suggests that osteotomy does not compromise subsequent knee replacement. However, the low quality of evidence precludes solid clinical conclusions.

## Background

High tibial osteotomy (HTO) is an accepted surgical treatment of medial unicompartmental osteoarthritis (OA) of the knee with varus mal-alignment in young patients. However, there is no sound evidence that an osteotomy is more effective than alternative non-operative therapies, such as valgus bracing or laterally wedged insoles [1]. Furthermore, results seem to deteriorate with time and this group of patients may require total knee replacement [2]. Success of primary total knee arthroplasty (TKA) with knee osteoarthritis is well established, and about 85% of patients are satisfied with the surgical outcome [3]. When considering osteotomy in the early treatment of medial compartment knee OA, subsequent TKA should not be compromised, and results should not deteriorate more rapidly than after primary TKA alone [4]. In the past, there have been reports of technical difficulties after failed HTO that influenced outcomes of knee replacement; however these studies were criticized due to patient selection bias [5,6].

The aim of this study was to collect the best available scientific evidence from clinical studies examining TKA after HTO compared with primary TKA, and determine whether an osteotomy influences clinical outcome after TKA. Although randomized controlled trials (RCT) are considered the ideal and highest level of evidence in making decisions about the care of individual patients, numerous "good" surgical practices have evolved into "standard of care" without being randomized against placebo or ineffective treatment options [7]. This probably explains why no RCT has been published on the effect of TKA with previous HTO or not, and that high-quality observational studies constitute the best available evidence [8]. We conducted a systematic review of non-randomized studies to analyze the effect of HTO on subsequent TKA, which may help facilitate the decision-making on performing osteotomy in the younger individual.

## Methods

### Identification of studies

A search of all relevant studies published in Medline and Embase up to September 2007 was performed to identify those investigating TKA after earlier HTO. The search strategy combined all phases of the optimal non-randomized studies strategy and used fixed method B, based on the study of Furlan et al. [9]. Key words used were: arthroplasty, replacement, knee, and osteotomy, and cohort studies (or controlled study, or follow-up studies, or prospective studies, or risk factors, or cohort.mp, or compared.mp, or groups.mp or multivariate.mp). Finally, all the references in the identified studies were checked to detect any additional published data.

Two reviewers (TR, MR) assessed the studies and whether they met the following inclusion criteria:

▪ patients in the study had TKA performed after prior HTO;

▪ the study had an observational design between 4 and 7 using the taxonomy of study designs described by Deeks et al. (controlled before-and-after, concurrent cohort, historical cohort, or case-control studies) [10];

▪ the article was written in English, German, or Dutch;

▪ full text was available for the article;

Disagreements on inclusion were resolved by discussion, and the final decision of a third reviewer (JV) was not necessary.

### Methodologic quality

Two reviewers (TR, MR) assessed the methodologic quality independently from each other. In order to avoid conflict of interest two other reviewers (RB, DM) re-assessed one study that was (co)-authored by TR and MR [11]. The critical appraisal of observational studies tool (Deeks) [10] and the methodological index for non-randomized studies (MINORS) form [12] were used. Disagreements were resolved in a consensus meeting. The maximum quality score was 12 for both forms. The measure of agreement between the two reviewers (TR, MR) is presented as kappa. The methodologic quality was used as an additional criterion for inclusion, and studies had to be of high quality to be selected for final review. High quality was based on a summary quality score, and defined as presenting an adequate concurrent cohort study that fulfilled 50% or more of the validity criteria on both quality instruments [13].

### Data extraction

Two reviewers (TR, MR) independently extracted the study characteristics and data on the intervention (operation time, lateral ligamental release, tuberosity osteotomy, tibial component insert), clinical outcome measures (postoperative knee range of motion (ROM) and clinical knee scores), and revision surgery (aseptic loosening, patellar loosening, deep infection, miscellaneous), using a pre-tested standardized form. Agreement on data extraction was reached by consensus.

### Evidence synthesis

The grade of evidence was determined following the guidelines of the GRADE (Grading of Recommendations, Assessment, Development, and Evaluation) working group [14]. GRADE acknowledges the primacy of RCT, but in addition recognizes circumstances in which high-quality observational studies generate high-quality evidence of treatment effects [15]. Grades of evidence are divided into the following categories: high, moderate, low, and very low; randomized trials are considered of high, observational studies of low, and any other evidence of very low quality. The similarity of estimates of effect across studies (consistency), and the extent to which people, interventions, and outcome measures are similar to those of interest (directness) may lower or raise the grade of evidence. We judged that quality of life of patients receiving knee arthroplasty will mostly be affected by knee function, pain, and adverse events such as aseptic loosening or infection, and considered postoperative ROM, postoperative knee scores and revision surgery as critical outcome measurements. The lowest quality of evidence for any of the outcomes was used for rating overall quality of evidence, as suggested by the GRADE working group. The data for this review were collected and analyzed in compliance with the procedures and policies set forth by the Helsinki Declaration.

## Results

### Included studies

Of the 458 articles identified using our search strategy, 17 met the inclusion criteria (Table 1) [4-6,11,16-28]. After the methodological quality assessment nine studies scored 50% or more on both quality scores and were included in this review: van Raaij [11]; Haslam [4]; Huang [16]; Karabatsos [17]; Meding [18]; Haddad [19]; Nizard [20]; Amendola [21] and Mont [22]. The mean score was 7.6 (range, 6 – 9) for the Deeks tool and corresponded with a 63% score. For the MINORS form the mean score was 7.1 (range, 6 – 8) and corresponded with a 59% score. The measure of agreement (kappa) between the two reviewers (TR, MR) was 0.86 for the Deeks tool quality score, and 0.95 for the MINORS form quality score. Disagreement occurred mainly because of reading errors and differences in interpretation of the comparability of group criteria.

**Table 1:** Identified observational studies reviewed for design and quality

		Design		Quality assessment	
	study	Year	Deeks classification	Deeks tool	Minors list
1	van Raaij [11]	2007	concurrent cohort (5)	9	8
2	Haslam [4]	2007	concurrent cohort (5)	8	7
3	Huang [16]	2002	concurrent cohort (5)	7	7
4	Karabatsos [17]	2002	concurrent cohort (5)	7	7
5	Meding [18]	2000	concurrent cohort (5)	8	7
6	Haddad [19]	2000	concurrent cohort (5)	8	7
7	Nizard [20]	1998	concurrent cohort (5)	8	7
8	Amendola [21]	1998	concurrent cohort (5)	6	6
9	Mont [22]	1994	concurrent cohort (5)	7	8

10	Parvizi [5]	2003	concurrent cohort (5)	5	9
11	Walther [23]	2000	concurrent cohort (5)	4	4
12	Toksvig-Larsen [24]	1998	concurrent cohort (5)	6	4
13	Bergenudd [25]	1997	concurrent cohort (5)	6	5
14	Gill [26]	1995	concurrent cohort (5)	5	4
15	Jackson [27]	1994	case-control (7)	3	2
16	Windsor [6]	1988	historical cohort (6)	3	4
17	Katz [28]	1987	concurrent cohort (5)	5	6

Those articles were selected with high quality on both instruments (≥ 50% or ≥ 6 points)

For the nine studies included, all studies had a follow-up matched (for at least three characteristics) pair comparison design. An overview of the characteristics is presented in Table 2. There were a total of 371 TKAs with previous HTO compared to 369 primary TKAs. A lateral closing wedge technique was used in four studies, one study presented results after valgus dome osteotomy, and four studies described combined or unknown osteotomy techniques. Osteotomy delayed TKA with a median of 7 (5 – 10) years. In one study patients served as their own controls when receiving bilateral knee replacement after unilateral HTO. One study presented two comparison groups; one was matched by pre-TKA deformity and the other by pre-HTO deformity. All populations, but one (59 years), had a mean age beyond 60 years at TKA surgery. Four studies contained more women than men; between 89 and 100% of patients were diagnosed with knee osteoarthritis. All studies reported on primary knee prosthesis designs, and the use of revision tibial components was not mentioned. Seven studies presented all cemented TKAs in almost all cases (94 – 100%). Only one study described a singular prosthesis design. Patella replacement was mentioned in four studies; in two studies all patients received patellar resurfacing, in one study about half of the patients, and in one study approximately 10% of the patients. The average follow-up after TKA was at least three years in all studies; with a median follow-up of 5 (3 – 13) years.

**Table 2:** Baseline characteristics of the 9 reviewed manuscripts

author, year		eligible knees, N	knees lost to follow-up, N	included patients, N	TKA, N	women, %	OA,%	time bought by HTO†, years	age at TKA†, years	follow-up†, years	patients with missing data, N
Van Raaij, 2007 [11]	HTO	18	4	12	14	83	100	4.8	60	3.7	0
	control	342	-	12	14	83	100	NA	61	4.0	0
Haslam, 2007 [4]	HTO	78	27	40	51	48	100	4.8	65	12.6	0
	control	-	-	44	51	48	100	NA	65	12.6	0
Huang, 2002 [16]	HTO	-	-	15	17	87	100	8	61	5	0
	control	-	-	14	17	86	100	NA	62	5	0
Karabatsos, 2002 [17]	HTO	-	-	20	22	50	95	8.4	59	5.2	3
	control	-	-	20	21	50	95	NA	60	4.7	3
Meding, 2000 [18]^a^	HTO	39	-	39	39	31	97	8.7	67	7.5	0
	control	39	-	39	39	31	97	NA	67	6.8	0
Haddad, 2000 [19]	HTO	50	0	42	50	62	100	7.3	65	6.2	2
	control	-	-	42	50	57	100	NA	66	-	1
Nizard, 1998 [20]	HTO	63	6	55	63	85	100	9.7	72	4.5	5
	control	537	-	-	63	78	100	NA	71	4.0	-
Amendola, 1998 [21]	HTO	42	-	39	42	36	100	5.4	64	3.1	-
	control	168	-	39	41	49	100	NA	65	3.1	-
Mont, 1994 [22]	HTO	80	7	73	73	49	89	5	62	6.1	0
	control I ^b^	974	-	73	73	49	89	NA	64	6.0	0
	control II ^c^	974	-	73	73	49	88	NA	64	6.2	0

NA = not applicable

- = not mentioned in manuscript

† = the values are given as the average

^a ^= patient group received bilateral TKA after unilateral HTO and served as own control

^b ^= matched for pre-TKA deformity (within 5°)

^c ^= matched for pre-HTO deformity (only varus knees)

### Study results

Intra-operative results are shown in Table 3. Four studies reported on operation time, which in three studies was significantly prolonged (median of 26 minutes) for patients receiving TKA after prior osteotomy (index group) compared with primary TKA (control group). In seven studies more lateral ligamental releases (median of 6) were necessary in the index group in comparison to the control group. Significant differences were found in two studies. Two studies found that more tibial tuberosity osteotomies were performed in the index group, and one of the studies noted a significant difference. No significant differences were reported in the distribution for thickness of the tibial inserts in two studies. The postoperative ROM (Table 4) was mentioned in six studies, and these studies detected less knee motion for the index group with a median of 10° (4° – 14°) in comparison to the control group. Two studies noted significant differences. All studies presented a knee score (Table 4) which contained pain and function evaluation; Hospital for Special Surgery score (HSS) in five studies, Knee Society clinical rating system score (KSS) in five studies, Western Ontario and McMaster University Osteoarthritis Index (WOMAC) in two studies, and the Baltimore knee score in one study. HSS and WOMAC scores were less favorable for the index group. All these differences, however, were not significant. Although the KSS knee score of the index group was lower in four out of five studies, only one study reported a KSS knee score significantly lower than the control group. The KSS function score of the index group was higher in three out of five studies, but no significant differences were found. One study used the Baltimore Knee score and detected a result in the index group significantly inferior to the control group. All studies but one reported on revision surgery after TKA (Table 5). In eight studies no significant differences between both groups were described for aseptic loosening, deep infection or other additional interventions. Seven studies reported on patellar loosening and found no significant differences between the index and control groups. One study commented on staged patellar re-surfacing for persistent patellofemoral symptoms, and described no differences between both groups.

**Table 3:** Intraoperative results for TKA with – compared to without prior HTO for the 9 reviewed manuscripts

author, year		TKA, N	operation time, minutes	lateral ligamental release	tibial tuberosity osteotomy	tibial component insert
van Raaij, 2007 [11]	HTO	14	120	3	1	10 mm
	control	14	115	0	0	8 mm
Haslam, 2007 [4]	HTO	51	-	-	-	-
	control	51	-	-	-	-
Huang, 2002 [16]	HTO	17	168 ^a^	5	-	-
	control	17	142 ^a^	0	-	-
Karabatsos, 2002 [17]	HTO	22	170 ^b^	7 ^c^	-	-
	control	21	118 ^b^	2 ^c^	-	-
Meding, 2000 [18]	HTO	39	-	4	-	-
	control	39	-	1	-	-
Haddad, 2000 [19]	HTO	50	23 min longer ^d^	22	-	difference; NS
	control	50	-	-	-	
Nizard, 1998 [20]	HTO	63	-	15 ^e^	7 ^f^	-
	control	63	-	1 ^e^	1 ^f^	-
Amendola, 1998 [21]	HTO	42	-	6	-	-
	control	41	-	2	-	-
Mont, 1994 [22]	HTO	73	-	-	-	-
	control	73	-	-	-	-

- = not mentioned in manuscript

^a ^p = 0.002, ^b ^p < 0.0001, ^c ^p = 0.0089, ^d ^p < 0.02, ^e ^p = 0.0001, ^f ^p = 0.03

NS = no significant difference after TKA with – compared to without prior HTO

**Table 4:** Postoperative outcome measures after TKA with – compared to without prior HTO for the 9 reviewed manuscripts.

author, year		patients, N	knee range of motion, °	HSS	KSS knee	KSS function	WOMAC	Baltimore knee score; excellent/good, %
van Raaij, 2007 [11]	HTO	12	110	79	79	70	NS	-
	control	12	120	82	90	80		-
Haslam, 2007 [4]	HTO	40	91 ^a^	79	-	-	-	-
	control	44	106 ^a^	80	-	-	-	-
Huang, 2002 [16]	HTO	15	-	-	83	75	-	-
	control	14	-	-	85	72	-	-
Karabatsos, 2002 [17]	HTO	17	-	-	-	-	NS	-
	control	17	-	-	-	-		-
Meding, 2000 [18]	HTO	39	113	-	89	81	-	-
	control	39	118	-	90	84	-	-
Haddad, 2000 [19]	HTO	40	93	87	91	70	-	-
	control	41	103	89	89	66	-	-
Nizard, 1998 [20]	HTO	50	101	79	74 ^b^	67	-	-
	control	-	105	83	81 ^b^	64	-	-
Amendola, 1998 [21]	HTO	39	101 ^c^	86	-	-	-	-
	control	39	115 ^c^	89	-	-	-	-
Mont, 1994 [22]	HTO	73	-	-	-	-	-	47 ^d,e^
	control I	73	-	-	-	-	-	64 ^d^
	control II	73	-	-	-	-	-	68 ^e^

Follow-up time on a median of 5 years.

HSS = Hospital for Special Surgery score (maximum 100 points)

KSS = Knee Society clinical rating system score (maximum 100 points)

WOMAC = Western Ontario and McMaster University Osteoarthritis Index

- = not mentioned in manuscript

NS = no significant difference after TKA with – compared to without prior HTO

^a ^p = 0.006, ^b ^p = 0.0001, ^c ^p < 0.005, ^d ^p < 0.01, ^e ^p < 0.001

**Table 5:** Revision surgery after TKA with – compared to without prior HTO for the 9 reviewed manuscripts.

author, year		TKA, N	aseptic loosening, N	patellar loosening, N	deep infection, N	other, N
van Raaij, 2007 [11]	HTO	14	0	0	0	2
	control	14	0	0	1	2
Haslam, 2007 [4]	HTO	51	4	1	0	0
	control	51	2	0	0	1
Huang, 2002 [16]	HTO	17	0	0	0	6
	control	17	0	2	0	4
Karabatsos, 2002 [17]	HTO	22	0	0	0	0
	control	21	0	0	0	1
Meding, 2000 [18]	HTO	39	1	3	0	2
	control	39	0	1	0	2
Haddad, 2000 [19]	HTO	50	1	-	1	11
	control	50	1	-	2	8
Nizard, 1998 [20]	HTO	63	0	1	0	13
	control	63	0	0	0	10
Amendola, 1998 [21]	HTO	42	0	1	0	3
	control	41	1	0	0	0
Mont, 1994 [22]	HTO	73	-	-	-	-
	control	73	-	-	-	-

Follow-up time on a median of 5 years.

- = not mentioned in manuscript

### Grade of evidence

No important inconsistencies among the nine studies were found in the direction of effect and the size of differences in effect; prolonged operation time, extra operative procedures, less postoperative knee ROM, and no increase of revision surgery was noticed for patients receiving TKA after prior HTO in the studies reflecting on the aforementioned outcomes. All studies described patients in their 6^th ^or 7^th ^decade of life receiving TKA because of symptomatic knee osteoarthritis. Knee replacement, regardless of prosthesis type, has more or less the same relative effects across most patients, therefore we judged the evidence obtained as direct [29]. Table 6 shows the overall quality assessment of the grade of evidence of the nine high-quality observational studies comparing TKA with – to TKA without prior HTO. We found no strong association among the studies and the overall quality of evidence, therefore, remained low.

**Table 6:** Quality assessment of the grade of evidence of the observational studies comparing TKA with – and without prior HTO

		Quality assessment					Summary of findings		
					No of knees		Effects		
No of studies	Design	Quality	Consistency	Directness	HTO	no HTO		Quality	Importance
Range of motion (6)									

	observational	no serious limitations	no important inconsistency	direct	251	251	4/6 studies no difference	low	critical

Knee clinical scores (9)									

	observational	no serious limitations	no important inconsistency	direct				low	critical
HSS (5/9)					212	212	5/5 studies no difference		
KSS (5/9)					175	176	4/5 study no difference		
other (3/9)					106	105	2/3 studies no difference		

Revision surgery because of (a)septic loosening (8)									

	observational	no serious limitations	no important inconsistency	direct	298	296	8/8 studies no difference	low	critical

## Discussion

Patients who require TKA for a failed HTO comprises a significant portion of those patients undergoing TKA [22]. Previous surgery may influence subsequent knee replacement, but so far, no guidelines considering TKA after prior osteotomy have been developed, and no grading of existing evidence has been determined. To our knowledge the present study is the first systematic review of existing literature on this topic. We used a limited search strategy in finding relevant non-randomized studies. Earlier Furlan et al. showed that the sensitivity of limited search strategies for a fixed set of controlled vocabulary and text words was between 95 and 100% [9]. We assessed the quality of the retrieved studies with established forms, and we found good interobserver agreement for both the Deeks tool and MINORS form (kappa 0.86 and 0.95; respectively).

Well-designed observational studies may provide high quality of evidence in circumstances described by the GRADE working group. The present study, however, could not raise the current low quality level of evidence. All studies presented relative small sample sizes, and pooling of the data would have provided a more precise association with the clinical outcomes. The heterogeneity of the studies, mainly due to differences in gender, osteotomy techniques, and time of follow-up, made quantitative pooling of the data impossible and a systematic review represented the best available method to synthesize the current literature [30]. This obviously limits the validity of the conclusions that can be extracted form this analysis.

Surgical methods have been recognized to be important factors in the longevity of knee implants [31]. Subperiosteal exposure of the proximal tibia and eversion of the patellar mechanism are more difficult in the post-osteotomy knee due to soft tissue scaring. Ligamentous imbalance may also compromise the implant procedure. Seven studies reported that more lateral ligamental releases were necessary for the post-osteotomy patients, and two studies found that more tibial tuberosity osteotomies were performed. These additional procedures may contribute to a significantly prolonged operation time for patients receiving TKA after prior osteotomy in three out of four studies. Many surgeons feel that intra-operative factors such as duration of the procedure may lead to inferior outcome after knee replacement. Earlier a logistic regression analysis showed that superficial infection was highly correlated with deep wound infection, which is a big threat to a successful outcome following knee joint replacement. Longer operating time, however, was no predictor of wound infection in 1181 patients undergoing TKA surgery [32]. Exposure difficulties and alterations in knee anatomy may compromise precision and accuracy of the surgical technique [31]. Especially tibial component fixation may be an issue after osteotomy due to the loss of metaphyseal bone stock. A revision tibial component with a canal-filling stem will increase the mechanical stability of tibial fixation [33]. On the other hand, a stemmed implant may prevent accurate placement of the tibial tray due to the asymmetric positioning of the medullary canal after HTO. Previous osteotomy may also influence patellar tracking leading to subluxation or rotatory instability. Malalignment and instability are major causes of early failure, and most revisions are performed within 5 years of primary arthroplasty [34]. After a median follow-up of 5 years we found no significant differences in TKA failure for the patients receiving TKA after previous osteotomy compared to primary TKA in all eight studies reporting on revision surgery. All studies presented in our review reported on primary knee prostheses, and did not describe the use of revision components. Earlier, a matched radiosteriometric study also showed no difference in failure rate after 10 years for primary knee components in patients with or without prior HTO [24]. Substantial improvements in the scores for physical health, such as those for pain and physical functioning seem to take place within the first 3 to 6 months after primary knee joint replacement, and studies with longer-term follow-up describe a lasting effect [35]. All six studies that discussed knee motion reported less range of motion with a median of 10° for patients receiving TKA after osteotomy compared to primary TKA patients. Two studies even noted significant inferior results. However, a multivariate analysis suggested that when determining the success of knee arthroplasty surgery ROM is far less important than overall function [36]. At mid-term follow-up this review could not detect any significant differences between both groups for overall function evaluated by standard knee clinical scores in eight out of nine studies.

Surgical treatment options for younger patients with unicompartmental OA of the knee remain controversial. Arthroplasty may have adverse effects. In an update study of data from the Swedish Knee Arthroplasty Register younger age was associated with an increased risk of prosthetic revision [37]. The cumulative revision rate for unicompartmental arthroplasty (UKA) was even higher than for TKA, and after removal of UKA loss of bone stock required significantly more osseous reconstructions in total knee revision compared with TKA after HTO [26,37]. One of the main reasons to perform HTO is delaying arthroplasty. The present review shows that the use of HTO postpones primary TKA for a median of 7 years in this subgroup of patients. This may be particular beneficial for patients with early onset knee OA, whose primary TKA might wear out before they die if they did not have the HTO.

## Conclusion

In summary our analysis represents the best available evidence on TKA after prior osteotomy, which seems to suggest that osteotomy does not compromise subsequent TKA. However, the overall low quality of evidence could not be raised by this review. Therefore, knee arthroplasty register data or multi-center high quality observational studies are needed to produce larger numbers and potentially generate higher quality of evidence to reach more solid conclusions.

## Competing interests

The authors declare that they have no competing interests.

## Authors' contributions

TR designed the study, acquired the data and drafted the manuscript. MR contributed substantially to the acquisition of data, carried out the statistical analysis and has been involved in interpretation of the data. AF and JV participated in the study design, and have been involved in critically revising the manuscript for important intellectual content. All authors read and approved of the final manuscript.

## Pre-publication history

The pre-publication history for this paper can be accessed here:


